# The immunobiology of myelodysplastic neoplasms: a mini-review

**DOI:** 10.3389/fimmu.2024.1419807

**Published:** 2024-09-16

**Authors:** Shruthi Kannan, Rolando A. Vedia, Jeffrey J. Molldrem

**Affiliations:** ^1^ Department of Hematopoietic Biology and Malignancy, Division of Cancer Medicine, The University of Texas MD Anderson Cancer Center, Houston, TX, United States; ^2^ Evolution of Cancer, Leukemia, and Immunity Post Stem cEll transplant (ECLIPSE), Therapeutics Discovery Division, The University of Texas MD Anderson Cancer Center, Houston, TX, United States; ^3^ Department of Stem Cell Transplantation and Cellular Therapy, Division of Cancer Medicine, UT MD Anderson Cancer Center, Houston, TX, United States

**Keywords:** myelodysplastic neoplasms, myelodysplastic syndromes, immune dysfunction, immunomodulatory therapies, immunobiology

## Abstract

This mini review summarizes the immunobiology of myelodysplastic syndromes, specifically focusing on the interactions between immune cells, cytokines, and dysplastic cells within the tumor microenvironment in the bone marrow. We elucidate in detail how immune dysregulation and evasion influence the initiation and progression of myelodysplastic syndromes, as well as resistance to therapy and progression to AML. In addition, we highlight a range of therapeutic strategies, including the most recent breakthroughs and experimental therapies for treating MDS. Finally, we address the existing knowledge gaps in the understanding of the immunobiology of MDS and propose future research directions, promising advancements toward enhancing clinical outcomes and survival for patients with MDS.

## Introduction

Myelodysplastic syndromes (MDS) are a complex and heterogeneous group of hematologic malignancies, characterized by dysfunctional hematopoiesis leading to cytopenias, reduced survival, and an increased risk of transformation to acute myeloid leukemias (AML) (1, 2). The annual age-adjusted incidence for MDS is 4 per 100,000 Americans, and increases substantially with increasing age, with median onset of 70 years (3). Additional risk factors for MDS include previous exposure to environmental toxins and disorders like autoimmune disorders or congenital predisposition syndromes like Fanconi anemia or germline variants in hematopoietic stem cells (HSCs) (1, 2). Depending on disease severity, MDS patients can be grouped into lower-risk and higher-risk groups. MDS patients can be categorized into lower-risk and higher-risk groups, depending on disease severity (1). Lower-risk MDS patients are much less likely to develop AML and have fewer mutations, lower myeloblast counts, with mutations in splicing factor genes such as *SF3B1*, that tends to improve survival. In contrast, higher-risk MDS patients carry more mutations, including *TP53* mutations which are associated with worse outcomes, more myeloblasts, and a higher risk of progression to AML (1, 2). The International Prognostic Scoring System (IPSS) and IPSS-R (Revised) categorize patients based on their risk level, percentage of myeloblasts, genetic abnormalities, and cytopenias (1). The Molecular International Prognostic Scoring System for Myelodysplastic Neoplasms (IPSS-M) was recently created to evaluate the prognostic and survival impacts of somatic mutations associated with development of MDS (4).

Despite three approved therapies and allogeneic stem cell transplantation (alloSCT), currently the only potentially curative strategy, the overall 5-year survival rate for MDS patients remains low at 31%. Moreover, not all patients are eligible for alloSCT, which is associated with increased morbidity and graft versus host disease (3). Hypomethylating agents (HMAs), particularly 5-azacytidine and 5-aza- 2’-deoxycytidine (decitabine), have been the standard of care for MDS patients, since their approval in 2004 and 2006, respectively. While some patients with MDS have shown clinical improvement with these agents, many do not respond positively to HMAs or experience a relapse after an initial positive response (5).

MDS can be caused by multiple molecular as well as non-molecular pathophysiologies. Mutations tend to accumulate in the HSCs of the bone marrow, which lead to uncontrolled proliferation, evasion of cellular apoptosis and thereby result in dysfunctional hematopoiesis and MDS (6). Patients usually present with a median of three somatic mutations, with 90% of patients exhibiting at least one. While several of these are driver mutations that determine prognosis and treatment choices, others have neutral or unknown prognoses (7). Interestingly, mutations in genes such as *SF3B1* are associated with better prognosis, while others like *TP53*, *DNMT3A* and *ASXL1* are associated with worse outcomes.

*TP53* mutations are of particular concern as they represent one of the highest-risk subsets of MDS patients with significantly reduced survival, complex karyotypes, heterogeneity in allelic states, drug resistance, and clinical outcomes (8, 9). Clonal dynamic modeling has shown that the persistence of these *TP53*-aberrant clones is the primary driver of malignant hematopoiesis and constitutes measurable residual disease (MRD), leading to relapse and treatment resistance (9).

An overview of the most mutated genes in MDS, their function, and immune connection is described in **Table 1**.

**Table 1:** presents a compilation of frequently mutated genes in MDS, along with their functions, the prevalence of mutations in MDS, their connection to the immune system, and corresponding references.

Gene	Introduction	Biochemical/Functional Role	Mutation in MDS	Immune Connection	References
**SF3B1**	SF3B1 is a crucial element of the encoded spliceosome U2 snRNP complex and identifies branch point sequences (BPS) to recruit and activate the spliceosome (10).	SF3B1 is linked to various physiological processes and metabolic pathways, such as erythropoiesis, iron metabolism, and DNA damage control (10). Mutations in SF3B1 can disrupt these pathways, alongside leading to DNA damage and increased inflammation, which may accelerate the development of MDS (10).	SF3B1 has almost exclusively heterozygous missense mutations that cause splicing abnormalities, and its high frequency in MDS occurs in HEAT domain repeat sequence codon 700 (K700E) (10).	MDS patients with SF3B1 mutations have elevated inflammatory cytokines, which impair normal hematopoietic function, resulting in a poor prognosis (10).	Cook et al. (2021) (7), Jiang, M. et al. (2023) (10).
**EZH2**	EZH2 regulates methylation of H3K27 and associated transcriptional repression as a member of the PRC2 complex (11).	EZH2, by affecting glucose, lipid, and amino acid metabolism, influences tumor cell metabolic patterns and cancer growth (12). Mutations in EZH2 drive cancer progression through these metabolic pathways. Additionally, tumor cell metabolism can impact EZH2 stability and methyltransferase activity (12).	EZH2 mutations in MDS and myeloproliferative neoplasms are typically loss-of-function mutations, correlating with poorer outcomes (11).	Some innate immune/inflammatory genes have promoter H3K27 methylation and increased mRNA expression with EZH2 mutation (11).	Cook et al. (2021) (7), Cabrero, M. et al. (2016) (11), Zhang, T. et al. (2020) (12).
**TP53**	TP53 encodes the tumor suppressor protein p53, which induces cell cycle arrest in response to different cellular stressors (7). TP53 mutations are the most widely mutated genes in all types of cancer.	TP53 affects cell cycle arrest, aging, DNA repair, apoptosis, autophagy, cell metabolism, ferroptosis, and reactive oxygen species generation (14). When the p53 pathway is impaired, cancer cells go through oncogenic signals and DNA damage, therefore leading to abnormal growth (14).	TP53 mutations are detected in 6-21% of MDS patients at diagnosis, predominantly as missense mutations. However, the extent of TP53 tumor suppressor activity loss varies considerably (7, 9, 13).	The TP53-mutated MDS patient group exhibits notable immune dysregulation compared to wildtype TP53, along with elevated expression of PD-L1 on LSC subsets (13).	Cook et al. (2021) (7), Patel et al. (2023) (8), Patel et al. (2021) (9), DiGennaro, J. et al. (2023) (13). Hernández Borrero, L. J. et al. (2021) (14).
**ASXL1**	ASXL1 is an epigenetic regulator that encodes a nuclear protein vital for maintaining gene homology and stabilizing gene expression across multiple loci (7, 15).	Mutations in ASXL1 have been linked to inflammasome signaling, which aids in activating NLRP3-dependent pyroptosis in MDS (16). ASXL1 has been demonstrated to be involved in both innate immunological and inflammasome signaling (16).	A truncated form of the ASXL1 protein, which impedes hematopoiesis, can result from mutations in the ASXL1 gene, usually close to the last exon (15).	Mutant ASXL1 has been implicated in modulating innate immunity by downregulating CLEC5 expression (a C-type lectin receptor activated by glycans), inhibiting myeloid differentiation. CLEC5 expression is notably diminished in MDS patients (15).	Cook et al. (2021) (7), Zhang, A. et al. (2022) (15), Barreyo, L. et al. (2018) (16)
**RUNX1**	RUNX1 encodes a transcription factor that prevents DNA from getting degraded, which vital for embryonic hematopoiesis, adult megakaryocyte, and platelet development, with most mutations leading to thrombocytopenia (7, 17).	RUNX1 reduces HIF-1α transcriptional activity to limit tumor angiogenesis and helps protect cells against oncogenesis (17). RUNX1 mutations are linked to increased illness and tumor progression in MDS and the removal of the DNA Damage response-mediated senescence barrier (17).	In therapy-related MDS, RUNX1 mutations are found in 8%–23% of cases and often in patients progressing to AML (7). These mutations, primarily located in the DNA-binding RUNT domain, typically result in the loss of RUNX1 function due to the removal of essential residues for binding (17).	Recent studies indicate that RUNX1 might be essential in modulating inflammation and autoimmunity (18). Notably, when Toll-like receptors 4 and 1/2 are activated, the inactivation of RUNX1 has been linked to an increase in the production of inflammatory cytokines from bone marrow cells (18).	Cook et al. (2021) (7), Kaisrlikova, M. et al. (2022) (17), Bellissimo, D. et al. (2020) (18).
**DNMT3A**	The DNMT3A methyltransferase gene plays a pivotal role in epigenetic gene regulation and *de novo* methylation.	It facilitates the addition of methyl groups to the cytosine residues of CpG dinucleotides, thereby shaping the epigenetic landscape and promotes HSC differentiation (7, 19). DNMT3A is thought to play a role in innate immune signaling (16).	DNMT3A mutations are frequently detected in 8-18% of MDS cases, primarily as heterozygous missense mutations, elevating the risk of disease progression (7, 19).	DNMT3A mutations have been linked to the modulation of innate immune signaling, characterized by heightened INFα/β levels and increased mortality rates. However, the precise mechanism remains unclear (16).	Cook et al. (2021) (7), Barreyro, L. et al. (2018) (16), Thol, F. et al. (2011) (19).
**IDH1/2**	The isocitrate dehydrogenase enzymes, IDH1 and IDH2, play a crucial role in cellular energy production by converting isocitrate to 2-ketoglutarate. Mutations in these enzymes are implicated in various cancers (7).	As components of the citric acid cycle, IDH1 and IDH2 help produce energy through the transformation of isocitrate into alpha-ketoglutarate (21). Mutations in IDH1/2 usually result in alpha-ketoglutarate being enzymatically converted to 2-hydroxyglutarate, an oncometabolite, in leukemias (21).	Mutations in IDH1 and IDH2 are relatively rare in MDS patients, however they have been associated with unfavorable disease progression (20). IDH2 mutations are found in 2.1%–4.0% of cases, while IDH1 mutations occur in 0.6%–3.6% of patients (7).	Patients with myelodysplastic syndrome (MDS) who have TET2/IDH mutations have been found to show a distinct phenotype in their natural killer (NK) cells (22). This suggests that these mutations are present in NK cells and are associated with changes in both phenotype and function (22).	Cook et al. (2021) (7), Thol, F. et al. (2010) (20), Ward, P. S. (2010) (21). Boy, M. et al. (2023) (22).
**TET2**	The TET2 gene, altered in hematologic malignancies, suppresses tumors and regulates gene transcription epigenetically to maintain normal hematopoiesis and stem cell biology (7, 22).	TET2 is associated with innate immune signaling (16). TET2 mutations increase NLRP3-dependent pyroptosis in MDS through inflammasome signaling (16).	TET2 mutations are common in MDS, found in 11%–33% of cases (7), and are linked to reduced survival post allogeneic HSCT (7). These mutations, mostly loss-of-function, hypermethylate genomic DNA in myeloid cells (22).	Loss of TET2 function increases innate immune signaling by downregulating immune cell proinflammatory cytokines and interferons (16). TET2 mutations in macrophages are linked to increased IL-1β-NLRP3 signaling, contributing to MDS (16). In MDS patients, TET2 mutations suppress NK-cell function (22).	Cook et al. (2021) (7), Barreyro, L. et al. (2018) (16), Boy, M. et al. (2023) (22).
**ETV6**	The ETV6 gene encodes a hematopoietic transcription factor that acts as a repressor in conjunction with SIN3A, NCOR, and HDAC3 complexes (7, 23). It is crucial to early bone marrow hematopoiesis (7).	In human hematopoietic cells, ETV6:P214L promotes overexpression of HDAC3-regulated interferon response genes and impairs the transcriptional repression of ETV6 (23). This alteration in inflammation increases the predisposition to leukemia (23).	ETV6 mutations are rare in MDS, appearing in only 1-3% of MDS cases. These mutations have been linked to a negative prognosis and unfavorable progression of the disease (7).	ETV6 dysfunction has been found to promote inflammation, adversely affecting thrombopoiesis and promoting leukemia development (23).	Cook et al. (2021) (7) Zhou, C. et al. (2022) (23).
**BCOR**	BCOR transcriptionally represses the HoxA gene cluster in myeloid cells and contributes to normal hematopoiesis and lymphoid cell development (7, 24).	The PRC1.1 complex, comprising BCOR, inhibits myeloid regulatory genes, guiding immune progenitors towards lymphopoiesis (24).	The loss of BCOR is hypothesized to provide MDS cells with a competitive edge regarding clonal growth (7). This mutation has been found in approximately 4%–6% of MDS patients (7).	BCOR loss-of-function mutations lead to B- and T-cell lineages having a selective disadvantage (24).	Cook et al. (2021) (7), Sportoletti, P. et al. (2021) (24).
**U2AF1**	The gene known as U2AF1, or small nuclear RNA auxiliary factor 1, is essential to the spliceosome pathway (7). It encodes an RNA-binding protein critical for recognizing pre-mRNA 3’ splice-acceptor sites (25).	U2AF1 participates in chronic innate immune signaling and stimulates the production of IRAK isoforms (25). The expression of the long isoform of the IRAK4 protein, IRAK4-L, is directly driven by U2AF1 mutations in MDS (25).	U2AF1 mutations occur in 5%–16% of MDS cases and are associated with poorer survival and a higher likelihood of leukemic transformation compared to wild-type patients (7).	Myeloid malignancies caused by U2AF1 mutations express “active” IRAK4 isoforms, which may be therapeutically targeted. In MDS and AML, this activation, in turn, triggers innate immune pathways (25).	Cook et al. (2021) (7), Smith, M. A. et al. (2019) (25).
**RAS**	The Ras superfamily of small GTPases, which consists of NRAS, KRAS, and HRAS, play a crucial role in various cellular processes, primarily governing cell proliferation, differentiation, survival, motility, and adhesion (7, 26). These genes commonly undergo mutations in different types of cancers.	The Ras superfamily of small GTPases controls numerous cellular functions as molecular switches (26). Receptor tyrosine kinases activate Ras via GEFs, loading it with GTP to activate downstream effectors. Ras activation activates Raf/Mek/Erk, PI3K, and RalGDS pathways (26).	Mutations in the RAS gene family are detected in 6.2%–8.8% of MDS cases, with NRAS mutations being the most prevalent (7, 27). These mutations impact overall survival in MDS (7).	Given the involvement of the Ras superfamily in numerous vital cellular processes, it holds a pivotal role in maintaining the proper functioning of the immune system (26). These molecular switches have several roles in regulating immune cell homeostasis and functions (26).	Cook et al. (2021) (7), Johnson, D. et al. (2012) (26) Buradkar, A. et al. (2020) (27).
**STAG2**	STAG2 controls sister chromatid separation during mitosis or meiosis as a part of the cohesin complex, which in turn impacts how cells divide (7).	Mutations of STAG2 have a major effect on cell division since it is involved in chromatin segregation during mitosis and DNA double-strand break repair (29).	5.9%–7.5% of MDS cases have STAG2 mutations, which have been linked to a lower survival rate regardless of other variables like age, gender, or IPSS (7).	STAG2 mutations are associated with a decrease in the expression of inflammatory genes and the loss of their expression (28). These mutations also result in an increase in populations of progenitor and stem cells with a decreased capacity for B-cell differentiation (28).	Cook et al. (2021) (7), Bhattacharya, S. et al. (2023) (28). Nasmyth, K. (2009) (29).
**CBL**	The Casitas B-lineage (CBL) lymphoma gene produces a tumor suppressor protein possessing E3 ubiquitin ligase activity, which functions as a downregulator of receptor tyrosine kinase activity and as an immune regulator (7, 30).	CBL-B accelerates the degradation of receptor-activated signaling proteins through ubiquitination (30, 31).	With mutations found in 1.5% to 5.1% of MDS cases, mutant CBL is thought to significantly impact the development of myeloid malignancies (7). While MDS patients with CBL mutations are not very common, their prognosis is worse than that of patients with wild-type CBL (7).	CBL ubiquitin ligases are known to play a role in maintaining immune quiescence and dendritic cell homeostasis (30). Immune dysregulation has been found to be significantly influenced by CBL-B deficiency (31).	Cook et al. (2021) (7), Tong, H. et al. (2021) (30), Janssen, E. et al. (2022) (31).
**SRSF2**	The SRSF2 gene produces serine/arginine-rich splicing factor 2, belonging to the serine/arginine-rich protein family. It participates in constitutive and alternative mRNA splicing, splice-site selection, and spliceosome assembly (7, 32).	During splicing, SRSF2 binds to exonic splicing enhancer (ESE) motifs in pre-mRNA via its RBD, facilitating exon recognition.	Mutations in SRSF2 are detected in 4%- 23% of MDS patients and are independently linked to elevated blast counts, higher rates of leukemic progression, and lower survival rates (7). SRSF2 mutations have a close association with *SRSF2* mutation with *RUNX1*, *IDH2*, and *ASXL1* mutations in MDS (33).	It has been demonstrated that SRSF2 mutations enhance NFκB activity and encourage the production of inflammatory cytokines in patient-derived cell lines and human and mouse myeloid cells (34).	Cook et al. (2021) (7), Masaki, S. et al. (2019) (32), Wu, S.-J. et al. (2012) (33), Pollyea, D. et al. (2019) (34).
**GATA2**	GATA2, a GATA transcription factor family member, is essential in regulating lymphatic angiogenesis and hematopoiesis. It regulates the growth and maturation of megakaryocytes and immature red blood cells (35).	GATA2 uses its zinc finger domains to control target genes and regulate hematopoiesis. It specifically plays a role in early embryonic hematopoietic specification (35).	GATA2 deficiency significantly elevates the lifetime risk of developing MDS, with an enhanced likelihood of progressing to AML (35). It is recognized as the predominant hereditary cause of MDS in adolescents with monosomy 7 (35). Furthermore, compared to wild-type cases, GATA2 mutant MDS is associated with lower survival and faster disease progression (7).	GATA2 mutation predisposition typically results in cellular immunodeficiency, leading to recurrent infections and autoimmune conditions. For individuals with GATA2-related MDS/AML, notable immune dysfunction often serves as the primary presentation (35).	Cook et al. (2021) (7), Kotmayer, L. et al. (2022) (35).
**ZRSR2**	ZRSR2, also known as U2 small nuclear ribonucleoprotein auxiliary factor subunit-related protein 2, is a gene found on the X chromosome and plays a role in the spliceosome pathway (7, 36). It is commonly mutated in myeloid malignancies.	ZRSR2 is involved in splicing and the spliceosome pathway, and the deficiency of ZRSR2 leads to impaired splicing of U12-introns, which leads to disease pathogenesis (36).	ZRSR2 mutations typically involve inactivation and are predominantly found in males. They are more common in MDS subtypes lacking ring sideroblasts and are linked to increased bone marrow blasts, reduced survival, and a higher likelihood of progressing to AML (36).	The absence of ZRSR2 in human cells has been shown to result in changes in differentiation potential towards erythroid and myeloid lineages, potentially leading to compromised immune responses (36).	Cook et al. (2021) (7), Madan, V. et al. (2014) (36).

High-resolution genome scanning and high-throughput sequencing have led to discovery of mutations in genes involved in methylation, transcription, cell signaling, histone modification, RNA splicing, and other pathways that may contribute to MDS progression, demonstrating that a better understanding of MDS genetics can target abnormalities and improve patient outcomes (37). Some of the non-molecular mechanisms which are implicated in the progression of MDS include the regulation of the immune system, the diversity of T-cells and their receptors, factors in the bone marrow microenvironment, abnormalities in methylation and other epigenetic markers, cell signaling molecules, and the length of telomeres (37).

Understanding the immune system’s key role in the pathogenesis of MDS is crucial, as it has a pivotal role in the development progression of the disease, and the risk of transformation to AML. The dysregulation and evasion of the immune system directly leads to impaired hematopoiesis and bone marrow failure, which is characteristic of MDS, shaping the microenvironment of the disease and influencing clinical responses such as resistance to HMAs. Studying the complex interaction of immune cells, cytokines and MDS stem cells, alongside elucidating disease mechanisms, also offers novel therapeutic targets for improving patient responses. MDS prevalence is expected to keep rising due to an aging population and the increasing number of people surviving chemotherapy or radiation treatment for cancer (4). Considering recent genomic, immunologic, and sequencing advances and the rising number of MDS cases, a comprehensive mechanistic approach is needed to understand MDS immune dysfunction. This mini review paper examines and condenses the immune components of MDS, encompassing pathophysiological mechanisms, treatment approaches, recent breakthroughs, and future research avenues, aiming to enhance understanding and guide future research towards improving patient outcomes and refining therapeutic strategies.

## Evidence for immune dysfunction in MDS

In recent years, increasing evidence has underscored the intricate interplay between immunological dysfunction and the pathogenesis of MDS, revealing insights into its multifaceted etiology and identifying potential targets for therapies.

It is becoming increasingly observed that immune-related occurrences are closely associated with MDS patients, such as cytopenias and autoimmune disorders that emerge before, during, or after MDS and bone marrow failure diagnosis (38). DNA-damaging treatments for autoimmune disorders, notably systemic lupus erythematosus (SLE) and rheumatoid arthritis (RA), have shown to increase the risk of MDS and other myeloid malignancies. Analysis of the molecular genetics of these MDS patients revealed that mutations in genes like *RUNX1*, *WT1*, *BCOR*, and *TP53* were associated with typical autoinflammatory symptoms, a higher likelihood of developing AML, lower survival rates, and a worse prognosis (39). Co-occurring mutations in *TET2*, *IDH1*, *IDH2* and *SRSF2* were linked to the development of systemic inflammatory and autoimmune diseases, some of which are also present in MDS patients (39).

Other rheumatologic and autoimmune disorders closely linked to MDS are VEXAS syndrome, leukocytoclastic vasculitis, pan vasculitis, autoimmune hemolytic anemia, and immune thrombocytopenia (38, 40). Patients with VEXAS syndrome, a monogenic disease caused by *UBA1* gene mutation, present with systemic autoinflammatory disease associated with MDS (41). Most vasculitis cases linked to MDS are idiopathic, and several cases have autoantibodies, which are frequently observed in low-risk MDS patients without excessive blasts (42).

The precise relationship between MDS and autoimmune disorders presents a significant and unresolved conundrum, raising questions about whether they co- occur, influence each other’s pathophysiology, or arise because of treatment for one another (38). Previous studies have focused on the prevalence of rheumatologic symptoms in MDS patients rather than on the role of autoinflammation in the development of MDS in patients with rheumatologic diseases. The factors that cause MDS’s autoimmune and rheumatologic symptoms warrant further studies to better understand its immune component.A less common form of MDS, hypocellular MDS, affects 10-15% of MDS patients and is characterized by a significant reduction in bone marrow cellularity (30%) (43). Compared to normal/hypercellular MDS, these patients are generally younger, exhibit more severe cytopenias, with higher transfusion dependence, and display lower bone marrow blast percentages (44). These patients also share cytogenetic abnormalities that suggest a common underlying pathogenesis, which might differ from the more cellular forms of MDS (45). Research indicates that using immunosuppressive therapies, particularly those utilizing anti-thymocyte globulin (ATG) to eliminate T cells, can be an effective method for treating this particular patient population (46). This indicates that the immunological characteristics of hypocellular MDS may significantly influence the mechanism and duration of treatment responses, similar to autoimmune conditions (47).

Hypocellular myelodysplastic syndrome (MDS) shares pathophysiological traits with nonmalignant bone marrow failure conditions like acquired aplastic anemia (AA) and paroxysmal nocturnal hemoglobinuria (PNH), which are linked to T cell-mediated autoimmunity and are associated with an increased risk of MDS and progressing to AML (48, 49). The transition from primary AA to secondary MDS can be indicated by the presence of PNH clones in hypocellular MDS (48). It is therefore very crucial to consider various immunological characteristics and features of hypocellular MDS, as they are likely to be pivotal in shaping disease progression.

Large granular lymphocytic leukemia (LGL) is a proliferation of cytotoxic T lymphocytes (CTL) that has been shown to co-exist alongside MDS clones. Likely due to a shared role of aging in these disorders, there seems to be a unique connection between MDS and LGL (50). Patients with both MDS/LGL show common MDS characteristics like abnormal cytogenetics, dysplasia of myeloid cells in the bone marrow, ringed sideroblasts and the existence of highly clonal somatic myeloid mutations (51). The evolution of MDS may be caused by repeated DNA damage due to excessive proliferation of large granular lymphocytes, immune suppression of myelopoiesis, and a myeloid escape clone (52).

Felty’s syndrome, characterized by a triad of RA, splenomegaly and granulocytopenia symptoms, has also been shown to co-occur with MDS and has been linked to increased incidence of MDS (53). The fact that alloSCT remains the only potentially curative regimen for MDS patients, suggests that one of the major components driving responses in MDS patients is the immune component.

While immune evasion mechanisms in MDS are not fully understood, the condition is associated with widespread immune cell dysfunction, as well as abnormal expression of checkpoints, cytokines, and inflammatory signaling pathways (54). Immune evasion in MDS is complex, varied, and dynamic, as evidenced by the coexistence and reciprocal transition of immune hyperfunction and immune repression (54, 55). The upregulation of immune checkpoints (e.g., PD-1/PD-L1, CTLA-4, TIM-3, TIGIT, CD47) on T cells in MDS creates an immune-evasive tumor microenvironment, leading to reduced cytotoxicity, impaired NK cell function, and T-cell exhaustion (54, 55). Abnormal levels of growth factors, chemokines, and cytokines, such as GM-CSF, TNF-α, TGF-β, IL-6, IL-8, IL-32, and IFN-γ, have been associated with immune evasion and worse outcomes in MDS (56, 57). Myeloid-derived suppressor cells (MDSCs) help tumor cells avoid immune detection by secreting immunosuppressive cytokines such as IL-10, IL-1β, and TGF-β, especially in high-risk MDS (58).

Several immune cells such as MDSCs, Tregs, NK cells, macrophages, monocytes and neutrophils also show abnormal regulation in MDS patients, including altered numbers, impaired function and abnormal cytokine secretion (55). The impaired ability of dendritic cells to stimulate T cells also contributes to the evasion of immune surveillance and progression to AML (55). The pathophysiology of MDS also involves persistent innate immunity and inflammatory signaling pathways, such as TLR signaling (55).

Overall, the increasing body of evidence about immunological dysfunction in MDS enriches our understanding of its causes and suggests the transformative capacity of immunomodulatory therapies in revolutionizing treatment for this complex disease.

### Treatments with immunomodulatory effects used to treat MDS

**Figure 1:**
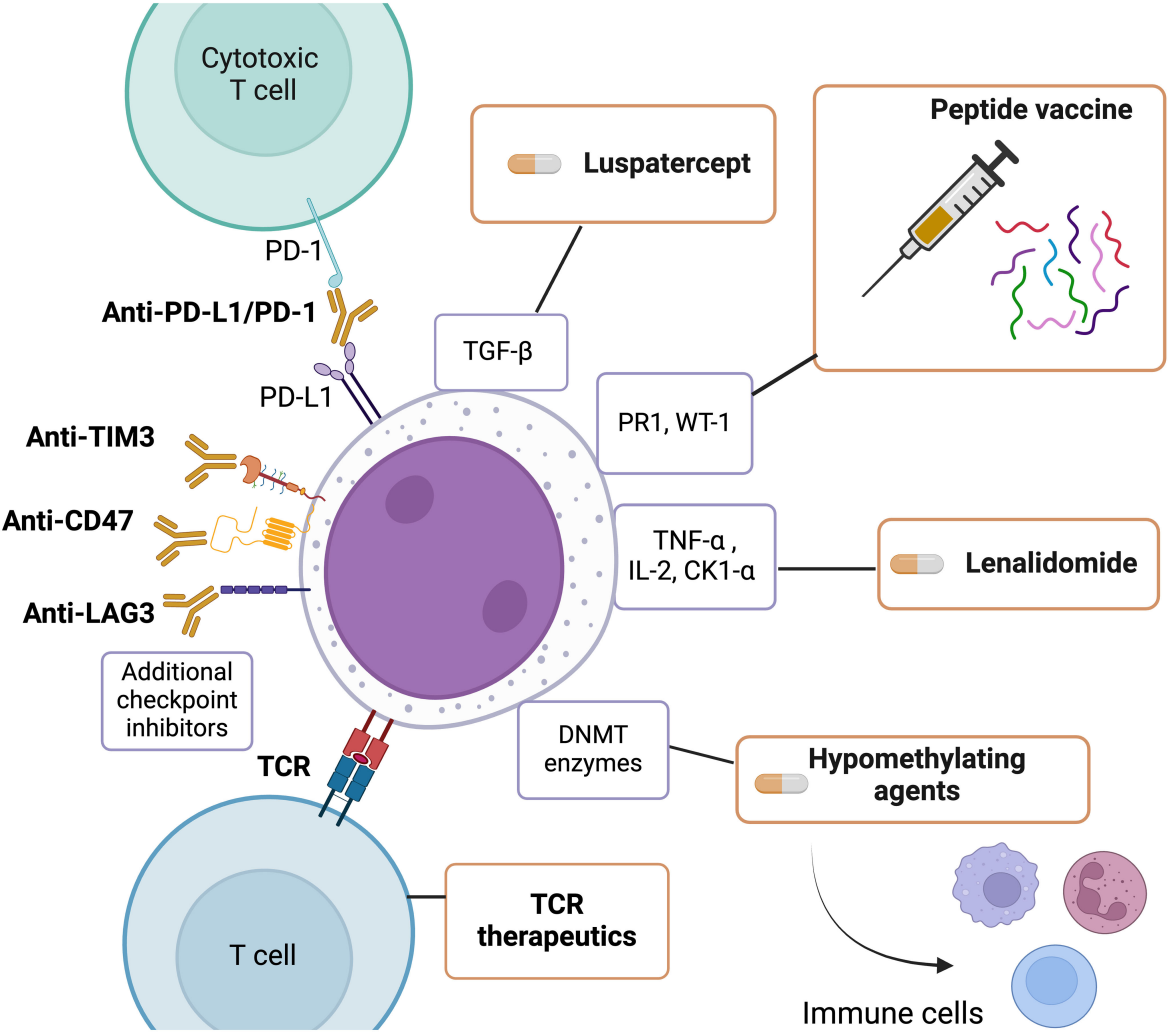
An overview of treatments commonly used to treat myelodysplastic syndrome with immunomodulatory effects. This Figure was created using BioRender.com.

With mounting evidence that the immune system plays a major role in MDS, ongoing research is prioritizing immune manipulation to improve treatment outcomes. Treatments for MDS that have immunomodulatory effects can be categorized into two groups: those that specifically target T lymphocytes and other cellular components of the immune system and those that modulate cytokines and their pathways to stimulate an immune response against malignant clones and myeloblasts. **Figure 1** describes an overview of treatments that have been commonly used to treat myelodysplastic syndromes that have been shown to have immunomodulatory effects.

### Targeting the cellular component of the immune system

Hypomethylating agents (HMAs) have been the first line of treatment for MDS for years as they reverse integrate into nucleic acids and inhibit DNMT enzymes, ultimately leading to DNA demethylation and reprogramming of tumor cells (59). HMAs increase non-self-recognition and cytotoxic T cell activity against cancerous cells (59). Some patients may initially respond to HMAs but can quickly acquire resistance. Patients with primary resistance to HMAs do not improve after 4-6 treatment cycles, while secondary resistance patients respond but eventually relapse. HMA resistance mechanisms are being studied but much still needs to be elucidated. However, it is evident that HMAs affect the immune system cells and the bone marrow niche (59). Previous research has shown that decitabine treatment increases CD38 expression on CD8+ and CD4+ T cells and is negatively correlated with reduced IFNγ production and T cell function (60). HMAs have also been shown to induce regulatory T cells (61) and have an activating effect on NK cells (62). Furthermore, studies have shown that decitabine can stimulate dendritic cells (63), and treatment with HMA reduces the presence of myeloid-derived suppressor cells (MDSCs) (64). HMAs have also been shown to boost anti-tumor immune responses by upregulating various genes associated with immunomodulatory pathways (65). Understanding how hypomethylating agents upregulate these genes can help identify MDS-expressed self-antigens in order to develop effective immune based treatments.

Likely due to high disease burden, low mutation burden, immunosuppressive tumor microenvironment, and moderate overexpression of immune checkpoint co-stimulatory receptors on T cells and ligands on leukemic cells, immune checkpoint therapies alone are ineffective in hematologic malignancies (65). Combination approaches of immune checkpoint inhibitors (anti-PD1/PD-L1 or anti-CTLA4 antibodies) in conjunction with HMAs are being explored in MDS (66–68). HMAs increase inhibitory checkpoint molecule expression, suppressing the immune response leading to T cell exhaustion, which can be reversed by PD-1/PD-L1 blockade (65). These combination therapies may also benefit from HMAs’ ability to overcome checkpoint inhibitor therapy’s immune evasion mechanisms, such as MHC downregulation, tumor antigen expression, and co-stimulatory ligand expression (65).

Several MDS clinical trials are testing additional immune checkpoint inhibitors like anti-TIM3, anti-CD47, and anti-LAG3 (69). Anatolia, an anti-TIM3 antibody, demonstrated clinical efficacy in Phase 1B trials involving higher-risk MDS patients when combined with HMA therapies (70). Magrolimab, an anti-CD47 antibody, has also shown synergistic effects when used in combination with azacytidine and other hypomethylating agents in these patients (71). PD-1H, a protein expressed by hematopoietic cells that acts as a homolog of PD-1 or PD-L1, and IRAK4, which is elevated in MDS patients with splicing factor mutations, are also potential immunological targets (69). In addition to immune checkpoint inhibition, adoptive cellular therapies such as bi-specific T-cell engager molecules (BiTEs), CAR-T, and CAR-NK cell therapies have shown minimal safety and efficacy in MDS patients (69). A deep comprehension of the interplay between patient immune response and MDS clones is needed to understand the lack of efficacy of these agents in MDS.

Peptide vaccines, such as the PR1 and WT1 antigen-specific combination, have been studied in a clinical trial for AML/MDS (72–74). Most patients exhibited a CD8+ T-cell response specific to PR1 or WT1, however, these vaccines have shown limited or transient efficacy. Advanced MDS stages are likely to be less conducive to eliciting an immune response through peptide vaccines. Optimal conditions may involve reduced tumor load after cytoreduction when the disease is in remission or post alloSCT (72).

In its early stages, T cell receptor (TCR) therapeutics are a developing focus in MDS research, requiring further study to enhance their effectiveness and safety in clinical settings. Initial research indicates that personalized adoptive cell therapy can select, immunize, and expand T cells outside the body against MDS patient-specific tumor cell neoantigens and neopeptides for therapeutic purposes.

It has been demonstrated that a subset of MDS patients (especially those that present with autoimmunity) recover from pancytopenia after receiving immunosuppressive therapies like ATG and cyclosporine (75). These interventions are especially successful in the early stages of MDS and can restore hematopoiesis and reduce transfusion dependence (75). Studies have shown that these cytopenias are caused by T lymphocytes suppressing hematopoietic progenitors, driven by predominant clonal T cells (75, 76). Effective hematopoiesis is recovered in patients who respond well to such immunosuppressive therapies, as evidenced by the loss of these clonally dominant T lymphocytes and the re-emergence of polyclonal T cells, which normalize the T-cell repertoire (76).

Several Toll-like receptor (TLR) signaling pathway components are elevated in MDS patients, and aberrant TLR signaling has been connected to ineffective hematopoiesis, a disease hallmark, and depletion of bone marrow CD34+ cells. As a result, drugs that block increased TLR signaling have been evaluated for possible therapeutic uses (77, 78). In leukemias, TLR agonists are also being studied for their possible anti-tumor effects, which include inducing cell death and differentiation by strengthening the immune system’s defense against leukemia cells (78).

### Targeting cytokines

MDS patients with chromosomal abnormality deletion 5q are treated with lenalidomide, an immunomodulator that impairs ribosomal proteins to induce cell apoptosis (79). In several clinical trials, MDS patients receiving lenalidomide achieved transfusion independence for at least 26 weeks, compared to placebo treated patients (80). Lenalidomide has been demonstrated to suppress the synthesis of pro-inflammatory cytokines, particularly TNFα, which is known to suppress hematopoiesis and act as an immune regulator (81). In this manner, lenalidomide acts as an immune regulator for hematopoiesis in MDS, alongside having direct effects on myeloid cells and lymphocytes by increasing IL2 (81). Lenalidomide has also been found to induce the ubiquitination of CK1α by the E3 ubiquitin ligase CUL4–RBX1–DDB1–CRBN (known as CRL4^CRBN^), which results in CK1α degradation. Since CK1α targets TP53 for degradation, lenalidomide can induce TP53 activation and growth inhibition (79). MDS del(5q) patients who develop resistance to lenalidomide tend to acquire *TP53* mutations. CK1α is also a negative regulator of Wnt signaling, and lenalidomide treatment also results in increased levels of β- catenin (79). Both these mechanisms lead to further disease progression.

Abnormal TGFβ signaling in MDS, characterized by reduced (inhibitory) I-SMADs and elevated TGFβ ligands like activin and GDF11, results in the activation of the TGFβ pathway, which impairs late-stage erythropoiesis consistent with morphologic erythroid dysplasia found in most MDS cases (82). Luspatercept, which decreases SMAD2 and SMAD3 signaling during erythropoiesis by binding to the TGFβ superfamily ligands, is a recently approved drug for MDS patients. Luspatercept is often given to MDS patients with *SF3B1* mutations or those with ring sideroblasts (83). A randomized, double-blind clinical trial found that luspatercept improved transfusion independence for at least 8 weeks compared to placebo treatment (84).

Other MDS therapies that have been investigated include the use of venetoclax, which targets the Bcl2 anti-apoptotic protein, in combination with 5-aza for relapsed/refractory MDS (85). Venetoclax caused a long-lasting response in the “CMP” pattern MDS, a HSC architecture consistent during HMA therapy and resistance. These studies suggest that molecular profiling may help treat MDS drug resistance (86). Due to elevated NEDD8 and NAE protein levels, in MDS patient samples, researchers have investigated using pevonedistat, a NEDDylation pathway drug, combined with 5-aza as a treatment option. This combination was well tolerated in elderly AML patients who were unfit for high-dose induction therapy, and it is now being targeted forHMA-resistant MDS patients (87–89). Several current clinical trials are investigating the effectiveness of signal transduction inhibitors like KRAS inhibitors and FLT3 inhibitors, pathways that are frequently altered in MDS (5). Several MDS patients have *IDH1/2* mutations, so isocitrate dehydrogenase inhibitors are being studied alone and with HMAs (5). These drugs cannot cure MDS because *IDH1/2* mutations tend to be subclonal but combining them with agents that target these mutations’ collaborative pathways may be beneficial (90). The effects on the immune component with these therapies needs to be further studied and characterized.

## Future directions

Various combinations of established and innovative targeted drugs, alone or with HMAs, have shown efficacy in randomized trials for MDS patients, with a significant associated immune component. However, the research for new and effective treatments for MDS has been hampered by various obstacles (4).

One major challenge in studying the immunobiology of MDS is the lack of robust mouse models and PDX models that accurately recapitulate disease features because primary human MDS cells do not grow well in xenograft mice (4). The recent creation of mouse models like MISTRG, allows better engraftment of patient derived MDS stem and progenitor cells (69). MDS also represents a spectrum of diseases, from low risk to high-risk features, and a single GEMM model is often insufficient in capturing all the features of this complex disease. Additional better validated mouse models will be needed to provide more reliable pre-clinical data for understanding the disease and developing innovative treatments (4).

Artificial intelligence and machine learning have facilitated the analysis of molecular abnormalities, pathological features, and clinical variables in biological sciences alongside advanced technologies such as long-read sequencing, immunopeptidomics, single-cell sequencing advancements, and differential expression analysis. These advancements can provide more accurate estimations of a patient’s survival and prognosis at any stage of their disease progression, incorporating a greater level of precision medicine into MDS alongside the integration of MDS assays (1, 2).

With these developments, MDS research will certainly benefit from an understanding of the pathophysiology and mechanisms of early-stage/lower-risk patients with few symptoms and a low mutational burden. Lower-risk MDS patients usually receive treatment with an emphasis on managing their symptoms, including anemia, neutropenia, thrombocytopenia, and other related conditions. Sequencing has shown that aberrant MDS cells in the immunophenotypically-defined HSC compartment can drive secondary AML progression. Furthermore, it has been observed that most HSCs are already mutated at the onset of MDS (5). Directing therapeutic interventions towards these specific cells may offer the optimal approach to enhance the prognosis of patients with MDS.

Similarly, future research should focus on hypocellular MDS patients and those with an autoimmune response. The immune microenvironment in MDS and the presence of antigen- specific T cell clones in MDS, particularly in immunoreactive cases, will also be better understood with the aid of advanced technologies. We can comprehend the antigens that T-cells in the MDS microenvironment encounter and react to when presented on HLA molecules, potentially leading to the discovery of novel neoantigens exclusive to malignant MDS cells. T cells may also have autoimmunity to myeloid precursors and contribute to cytopenia’s, which needs to be further studied.

The bone marrow microenvironment’s MDSC population should also be studied alongside T cells. MDSCs regulate most cancers’ immune systems, so it is important to study their role in the bone marrow microenvironment and distinguish them from other myeloid elements to understand their unique role in MDS (91).

Intensive focus on MDS neoantigen research in high-risk or advanced-stage MDS patients could be particularly advantageous, as tumors with higher mutational burden are typically more responsive to T cells (92). Regarding this, a deeper comprehension of the dysregulation of antigen presentation in MDS may help us figure out how to enhance the accuracy of target and therapeutic antigen presentation.

In conclusion, the intricate and multifaceted nature of MDS demands a multipronged investigative approach that delves deeper into the underlying mechanisms driving these disorders. Mechanism-based studies in clinical trials that characterize immune effects on myelodysplastic clones or precursors need to be carried out, as mounting evidence suggests that immune dysregulation plays a pivotal role in driving pathogenesis and progression of MDS. Collaborative efforts to target the immune component hold promise for novel therapeutic strategies aimed at altering the natural history of MDS, offering renewed hope for improved outcomes and quality of life of affected individuals.
